# EVALUATING AN INTERVENTION TO PREVENT SELF-NEGLECT IN VULNERABLE ADULT HEALTHCARE PATIENTS

**DOI:** 10.1093/geroni/igz038.3359

**Published:** 2019-11-08

**Authors:** Miriam Rose, Farida Ejaz, Courtney Reynolds, Minzhi Ye

**Affiliations:** Benjamin Rose Institute on Aging, Cleveland, Ohio, United States

## Abstract

Alleged self-neglect is the most commonly reported type of abuse to Adult Protective Service (APS) agencies nationwide. Researchers, healthcare practitioners and APS staff in Texas collaborated on a project funded by the U.S. Administration for Community Living to develop and evaluate an intervention to prevent self-neglect in older and/or disabled adults. Nineteen primary care clinics in a large healthcare system were randomized to intervention (intensive case management over a four-month period) and control (usual healthcare) groups. Patients with risk factors for self-neglect in these clinics were randomly selected, and 480 patients consented to participate in the study. Baseline EMR data indicated the most common risk factors for self-neglect included depression (54% of these participants), dependence in activities of daily living (28%), and dementia (27%). Social workers conducted a home visit with 287 intervention clinic patients, identified their needs, developed a care plan, and followed up regularly with patients. Based on the Adult Self-Neglect Assessment, 61% were identified as having concerns related to self-neglect. Their most frequently identified areas of need for help were food assistance/nutrition (54%), functional limitations (40%), social isolation (36%), home modifications (34%), and mental health issues (32%). Along with public assistance, and referrals to home- and community-based services, findings are promising for preventing patients from becoming self-neglecting in the future. Only 7 intervention clinic participants were reported by project staff to APS for suspected abuse, neglect or exploitation during the 16-month study. This type of multi-disciplinary collaboration can inform development of evidence-based innovations in practice and policy.

